# Joint FAO/IAEA Coordinated Research Project on “*Mosquito Handling, Transport, Release and Male Trapping Methods*” in Support of SIT Application to Control Mosquitoes

**DOI:** 10.3390/insects14020108

**Published:** 2023-01-19

**Authors:** Maylen Gómez, Brian J. Johnson, Hervé C. Bossin, Rafael Argilés-Herrero

**Affiliations:** 1 Insect Pest Control Subprogramme, Joint FAO/IAEA Programme of Nuclear Techniques in Food and Agriculture, P.O. Box 100, 1400 Vienna, Austria; 2 QIMR Berghofer Medical Research Institute, Brisbane, QLD 4006, Australia; 3 Institut Louis Malardé, William A. Robinson Polynesian Research Center, Medical Entomology Laboratory, P.O. Box 30, 98713 Papeete, Tahiti, French Polynesia

Mosquito-borne diseases are among the most important public health problems worldwide. In recent years, mosquito-transmitted dengue has become a major international public health burden due to the increasing spread of invasive mosquito species of the genus *Aedes,* which also transmit chikungunya, Zika, and yellow fever. The failure of traditional insecticide applications to control the increasing threat of dengue [1] and other mosquito-borne diseases, such as the 2015–2016 pandemic spread of Zika virus to the Americas, the Caribbean, and Africa, have renewed demands to develop sustainable, environmentally friendly, and innovative approaches to mosquito control [2]. In response to this challenge, new methods involving the rearing and release of large numbers of male mosquitoes to eliminate or suppress local *Aedes* populations are being developed [3]. These methods include the use of the sterile insect technique (SIT) to control major disease-transmitting mosquito species to reduce the global public health burden of mosquito-borne diseases [4].

The SIT represents an environmentally friendly alternative to area-wide integrated pest management (AW-IPM) programs with a potential application for use against mosquitoes. In the last two decades, the Joint FAO/IAEA Programme of Nuclear Techniques in Food and Agriculture, particularly its Insect Pest Control Subprogramme, in collaboration with its Member States, has developed and evaluated this technology as a tool for controlling mosquito vector populations as a component of AW-IPM. The SIT requires the production, sterilization, handling, transportation, release, and field monitoring of millions of male mosquitoes. The maximization of efficiency and insect fitness at each stage of the SIT processes is critical to maintaining male sexual competitiveness and, ultimately, programmatic success. Methods for population monitoring pre- and post-release are also essential to program planning, implementation, and assessment. The definitive goal is to determine the effectiveness of the developed SIT package in suppressing the target vector population. The viability of mosquito-based SIT packages is currently being evaluated and success has been demonstrated in several pilot projects, yet technological challenges remain.

Many of the remaining technological challenges regarding the efficient and economical delivery of SIT packages for mosquitoes relate to the development and optimization of handling, transportation, and release procedures. The optimization of each component must be achieved with the explicit goal of maximizing sterile male survival, dispersion, and sexual performance in the field. Similar efforts must also be put forth to develop affordable and efficient surveillance tools to improve the monitoring of target vector populations before, during, and after the release of sterile males. In light of these demands, in 2014, the FAO/IAEA convened a consultant meeting to prepare a proposal for a coordinated research project (CRP) on “*Mosquito Handling, Transport, Release and Male Trapping Methods*”, in support of the SIT application for mosquito control. This CRP was approved and initiated in 2015 and involved the participation of 22 scientists from 19 Member States, many of which had been heavily affected by the public health and economic impacts of mosquito-borne diseases [5,6]. The main objective of this CRP was to provide the necessary technical advances in the handling, release, and subsequent monitoring of male mosquitoes to enable the cost-effective application of the SIT against mosquitoes to reduce the burden of mosquito-borne diseases in affected Member States. For this purpose, CRP-sponsored research was undertaken to determine the optimal conditions for the chilling, marking, packaging, and transportation of sterile male mosquitoes. The CRP also endeavored to explore different approaches to the release of sterile male mosquitoes in a controlled, traceable, and documented manner over large urban and rural areas, with the ability to target specific areas whilst maintaining a high quality of released insects. Lastly, the CRP aimed to promote advances in male-based population monitoring tools for use in the evaluation of an AW-IPM program with an SIT component to improve the field evaluation of sterile male mosquito performance and operational impact of the SIT.

The research carried out in the framework of this CRP generated key achievements in areas relevant to the SIT application to control mosquitoes. Among these outcomes, particular mentions must be given to the following: (1) Novel and efficient self-marking techniques were established to improve our understanding of male movement, competitiveness, survival, and potential interaction with closely related species post-release [7,8]. As the next step, these new marking systems will be upscaled and validated under operational conditions in SIT field projects targeting the *Aedes* species. (2) One of the techniques evaluated included the novel large-scale marking of male mosquitoes via administration of Rhodamine B via sugar feeding. The mentioned technique brings the advantage of marking all of the tissues of the mosquitoes, including sperm and seminal fluid, such that the mark can be retrieved and identified in mated females. Thus, the use of Rhodamine B enables researchers to estimate the sexual competitiveness of sterile male mosquitoes under field conditions [9,10]. (3) Additionally, the impacts of temperature, time, and compaction, and the interactions between them, were explored to improve the handling, shipping, and transportation of chilled males [11]. Based on these results, suitable protocols for short- and long-distance shipments were developed for *Aedes aegypti* [12,13], with likely extension to other members of the Stegomyia subgenus, including *Aedes albopictus* and *Aedes polynesiensis*. Countries such as Brazil have already implemented and evaluated developed protocols under operational conditions [13]. (4) Alongside these protocols, methods for ground and aerial releases (using drones) were developed and evaluated. Remotely piloted aircraft systems (RPASs) with embedded mosquito release devices have been developed and evaluated under laboratory and field conditions for *Ae. aegypti* in countries such as Mexico [14]. While there are still several challenges to be addressed before implementing this approach in SIT pilot projects against mosquitoes, results from CRP-conducted research demonstrate that drone-based aerial releases are a powerful and cost-effective alternative to traditional ground-based releases. (5) To better assist all forms of release, a novel all-in-one release container was developed to maximize release densities while maintaining male viability [15]. The developed container allows mosquitoes to be maintained in relatively undisturbed conditions from the pupal stage until release as adults, to eliminate the negative effects of post-emergence handling associated with the majority of large-scale release systems. (6) Lastly, the behavioral responses of male *Ae. albopictus* to different volatile compounds were studied to identify chemicals which may enhance male *Ae. albopictus* surveillance [16]. Decanoic acid was found to be attractive to male *Ae. albopictus* in moderate concentrations, whereas most other compounds were found to act as repellents. Studies such as this are critical to developing alternative surveillance strategies to assist mosquito-based SIT packages.

During the CRP, four general meetings were held, spaced approximately 18 months apart, in which the CRP’s participants reported and shared relevant research findings, challenges, and solutions, and formalized their forthcoming research plans and objectives. Overall, the successful CRP implementation resulted in new knowledge, tools, and protocols for the monitoring, marking, handling, transportation, and release of sterile male mosquitoes to support mosquito-based SIT packages. Accordingly, the current Special Issue (SI) has compiled ten papers reporting results in the areas outlined above. Alongside these results, the next step is to transfer these new tools and procedures to Member States to benefit mosquito SIT pilot trials worldwide. As with any SIT application, success ultimately depends on the ability of released males to effectively compete with wild males for available females. An SIT pilot project, and a later program on an operational scale, will require several million adult male mosquitoes to be produced, transported, and released into target sites, after being previously sterilized, marked, chilled, and compacted for easy handling and packing, all with the goal of maintaining male competitiveness post-release. Therefore, the guest editors are confident that the compiled information in this SI is relevant and extremely useful for ongoing SIT pilot projects and the eventual scale-up of the technology to meet the demands of AW-IPM programs.

**Supplementary Materials:** This article has been published as part of Insects 13, 2022, Special Issue on “*Mosquito Handling, Transport, Release and Male Trapping Methods*”. The full contents of the supplement are available online at https://www.mdpi.com/journal/insects/special_issues/Mosquit_Handling_Transport_Release_and_Male_Trapping_Methods.

## Data Availability

Not applicable.
